# Neural representations of anxiety in adolescents with anorexia nervosa: a multivariate approach

**DOI:** 10.1038/s41398-023-02581-5

**Published:** 2023-08-15

**Authors:** René Seiger, Nicco Reggente, D.S.-Adnan Majid, Ronald Ly, Reza Tadayonnejad, Michael Strober, Jamie D. Feusner

**Affiliations:** 1 General Adult Psychiatry and Health Systems, Centre for Addiction and Mental Health, Toronto, ON Canada; 2 Institute for Advanced Consciousness Studies, Santa Monica, CA USA; 3 Semel Institute for Neuroscience and Human Behavior, University of California Los Angeles, Los Angeles, CA USA; 4 Department of Psychiatry and Biobehavioral Sciences, University of California Los Angeles, Los Angeles, CA USA; 5 Division of Neuromodulation, Semel Institute for Neuroscience and Human Behavior, University of California, Los Angeles, Los Angeles, CA USA; 6 Division of Humanities and Social Sciences, California Institute of Technology, Pasadena, CA USA; 7 Department of Women’s and Children’s Health, Karolinska Hospital, Karolinska Institutet, Stockholm, Sweden; 8 Department of Psychiatry, University of Toronto, Toronto, Canada

**Keywords:** Neuroscience, Psychiatric disorders

## Abstract

Anorexia nervosa (AN) is characterized by low body weight, fear of gaining weight, and distorted body image. Anxiety may play a role in the formation and course of the illness, especially related to situations involving food, eating, weight, and body image. To understand distributed patterns and consistency of neural responses related to anxiety, we enrolled 25 female adolescents with AN and 22 non-clinical female adolescents with mild anxiety who underwent two fMRI sessions in which they saw personalized anxiety-provoking word stimuli and neutral words. Consistency in brain response patterns across trials was determined using a multivariate representational similarity analysis (RSA) approach within anxiety circuits and in a whole-brain voxel-wise searchlight analysis. In the AN group there was higher representational similarity for anxiety-provoking compared with neutral stimuli predominantly in prefrontal regions including the frontal pole, medial prefrontal cortex, dorsolateral prefrontal cortex, and medial orbitofrontal cortex, although no significant group differences. Severity of anxiety correlated with consistency of brain responses within anxiety circuits and in cortical and subcortical regions including the frontal pole, middle frontal gyrus, orbitofrontal cortex, thalamus, lateral occipital cortex, middle temporal gyrus, and cerebellum. Higher consistency of activation in those with more severe anxiety symptoms suggests the possibility of a greater degree of conditioned brain responses evoked by personally-relevant emotional stimuli. Anxiety elicited by disorder-related stimuli may activate stereotyped, previously-learned neural responses within- and outside of classical anxiety circuits. Results have implications for understanding consistent and automatic responding to environmental stimuli that may play a role in maintenance of AN.

## Introduction

Anorexia nervosa (AN) is characterized by restricted caloric intake and is associated with low body weight, a fear of gaining weight or becoming fat, and, often, a distorted body image [1]. It typically onsets in adolescence and females are most commonly affected [2]. The excessive food restriction and the resultant medical manifestations of malnutrition, as well as suicidality, make it one of the deadliest psychiatric diseases, and has a high rate of relapse [3, 4].

Although the symptomology is well-described, the etiology is still not well understood; theories include a combination of genetic and biological causes, environmental factors, and personality traits [5]. Comorbidities are common in AN, such as obsessive-compulsive disorder (OCD) [6], body dysmorphic disorder (BDD) [7], and, particularly, anxiety disorders [8, 9], which have lifetime prevalence estimates as high as 55–83% [10, 11]. Anxiety and anxiety disorders are typically present in children and adolescents even before the diagnosis of AN [10, 12, 13]. Moreover, anxiety in AN significantly affects treatment outcomes and lowers the probability of recovery [14, 15]. Thoughts about food and calories as well as body appearance are frequent [16] and are typically connected to feelings of anxiety and discomfort. Aberrant responses to such stimuli in anxiety brain circuits may therefore play a role in the illness [17]. Repeated exposure to anxiety- or fear-eliciting stimuli in the context of, for example, food, eating, weight, and body image, could shape brain circuits, leading to learned, enduring, and “engraved” anxiety brain response patterns [18].

The neural basis of anxiety in those with AN has been investigated using neuroimaging, including several studies with mainly young female adults (fewer such studies have been conducted in adolescents), focused on responses to aversive food or body-related stimuli and which may have evoked anxiety or fear/threat perception. One such study using food stimuli, which were experienced as threatening or aversive by the participants with AN, demonstrated increased activity in the medial orbitofrontal cortex (mOFC) and regions within the anterior cingulate cortex as well as decreased activation in the lateral prefrontal cortex, cerebellum, and inferior parietal areas, in contrast to the control group [19]. Another study utilizing a similar approach detected increases in the amygdala and reductions in the midcingulate cortex in those with AN compared to the healthy comparison group [20]. When people were presented with pictures of food and instructed to think about eating these food items, higher activation in the visual cortex and prefrontal regions and decreases in cerebellar areas in AN participants were found [21]. A study by Horndasch et al. looked at responses to high-caloric as well as low-caloric food stimuli in adolescents and young adults with AN. For the high-caloric condition, adolescents with AN compared with healthy individuals demonstrated higher activation in inferior frontal and medial prefrontal areas as well as in the anterior insular cortex and lower activation in the cerebellum, while the results for young adults were less clear [22]. A similar study of calorie-relate fear was investigated by contrasting high vs low calorie drinks; participants with AN showed stronger activity in comparison to the control group in limbic and paralimbic regions including the insula, anterior cingulate cortex, and the amygdala-hippocampal area [23]. A study using negative words related to body image revealed greater activation in the amygdala in patients with AN when negative words were contrasted against neutral ones [24]. Another investigation comprising adolescent females did not find differences between the group with AN and healthy controls in a social evaluation task, but showed that levels of anxiety correlated with neural activity within regions of the medial prefrontal cortex and the cingulate [25]. In sum, these studies suggest generally high neural activation involving fronto-limbic anxiety network regions, activation extending beyond these systems such as in parietal and visual cortical regions, and deactivations within cerebellar regions.

However, these studies were carried out using mass-univariate approaches. Multivariate approaches [26, 27], on the other hand, such as representational similarity analysis (RSA) [28], provide additional information by delivering insights into distributed patterns across the brain [29–31]. Those with AN typically suffer from anxiety symptoms, which are regularly triggered day-to-day by reminders of food, calories, and body image. This suggests the possibility of aberrantly consistent responses to those stimuli, which could be reflected in highly consistent anxiety circuit (and possibly extended beyond anxiety circuit) brain activation patterns. To test this, we used a multivariate RSA approach to contrast anxiety processing between adolescent female participants with AN and a non-clinical comparison group with mild anxiety, and to examine associations between anxiety and multivariate brain responses, using anxiety-evoking and neutral (control) words. Enrolling a cohort with mild anxiety allowed us to both examine group comparisons between AN and non-clinical population as well as testing for dimensional associations with anxiety across the sample. We hypothesized that groups would differ significantly in terms of higher consistency of neural responses in anxiety circuits in the AN group, as reflected by greater representational similarity trial-by-trial across anxiety stimuli. In addition, we hypothesized that a whole-brain analysis would reveal higher consistency of neural responses (higher representational similarity) in extended regions involved in anxiety and processing of emotional stimuli. Higher representational similarity would suggest a more consistent, coordinated multivariate response, which could reflect longstanding patterns of repeated activation and perhaps more highly conditioned and persistent responses, as demonstrated previously in a conditioning experiment analyzed using RSA [18].

To accomplish this, we conducted two main analyses. First, a region of interest (ROI) approach was utilized involving the following anxiety circuit regions, which were combined into an overall ROI: amygdala, anterior cingulate cortex (ACC), insula, medial prefrontal cortex (MPFC), ventral tegmental area (VTA), and the bed nucleus of the stria terminalis (BNST) [32–36]. Second, we conducted a whole-brain searchlight analysis [37]. For the whole-brain analysis, differences in RSA values were expected in both the anxiety circuit regions described in the ROI approach as well as other areas associated with anxiety processing: ventrolateral and dorsolateral prefrontal cortex, striatum, hippocampus, uncus, inferior parietal, postcentral gyrus, precuneus, ventral visual stream, and other temporal regions, and the cerebellum [38–45]. To assess dimensional associations between brain representational similarity patterns and anxiety symptoms as well as reported state anxiety, Hamilton Anxiety Rating Scale (HAM-A) [46] scores and individual anxiety ratings after the scan were correlated with the results from the searchlight and ROI analyses, across participants. Correlations were conducted for the anxiety word task condition, as well as the neutral word condition as a control. We predicted significant correlations between both anxiety symptoms and state anxiety with the RSA values, which would not be observed for the neutral word condition. The hypotheses were pre-registered: https://aspredicted.org/59P_HLT (A divergence from the pre-registered hypotheses was that we chose to test subjective state anxiety ratings after rather than before the scan, as the former would better approximate state anxiety during the experiment).

## Material and methods

### Participants and clinical evaluations

Females between the ages 10–19 were recruited for the AN and comparison group from all ethnic and racial backgrounds, in the greater Los Angeles area. This study was approved by the UCLA Institutional Review Board and written informed consent was obtained from the participants and, for those younger than 18, their parents or legal guardians. Participants with AN met DSM-5 criteria for restricting type anorexia nervosa within the recent 6 months and were recently treated intensively in an inpatient, residential, or partial hospitalization program. Non-clinical comparison participants were included if they did not meet current criteria for any DSM-5 disorder and did not take any psychiatric medications. However, scores on the anxiety subscale of the Depression Anxiety Stress Scale (DASS-21) [47] had to be at least 0.5 standard deviations higher than population norms, in the interest of planned dimensional analyses of anxiety across all participants. Anxiety experienced during the last week before study inclusion was measured with the Hamilton Anxiety Rating Scale (HAM-A). In addition, all participants were asked after each scan to rate their level of anxiety on a Likert scale from 0 to 10 by answering the question: “What is the general level of anxiety you're feeling right now?” Details regarding study participants and evaluations can be found in the supplemental material. The study was registered on Clinicaltrials.gov NCT02948452.

### FMRI paradigm

**Fig. 1:**
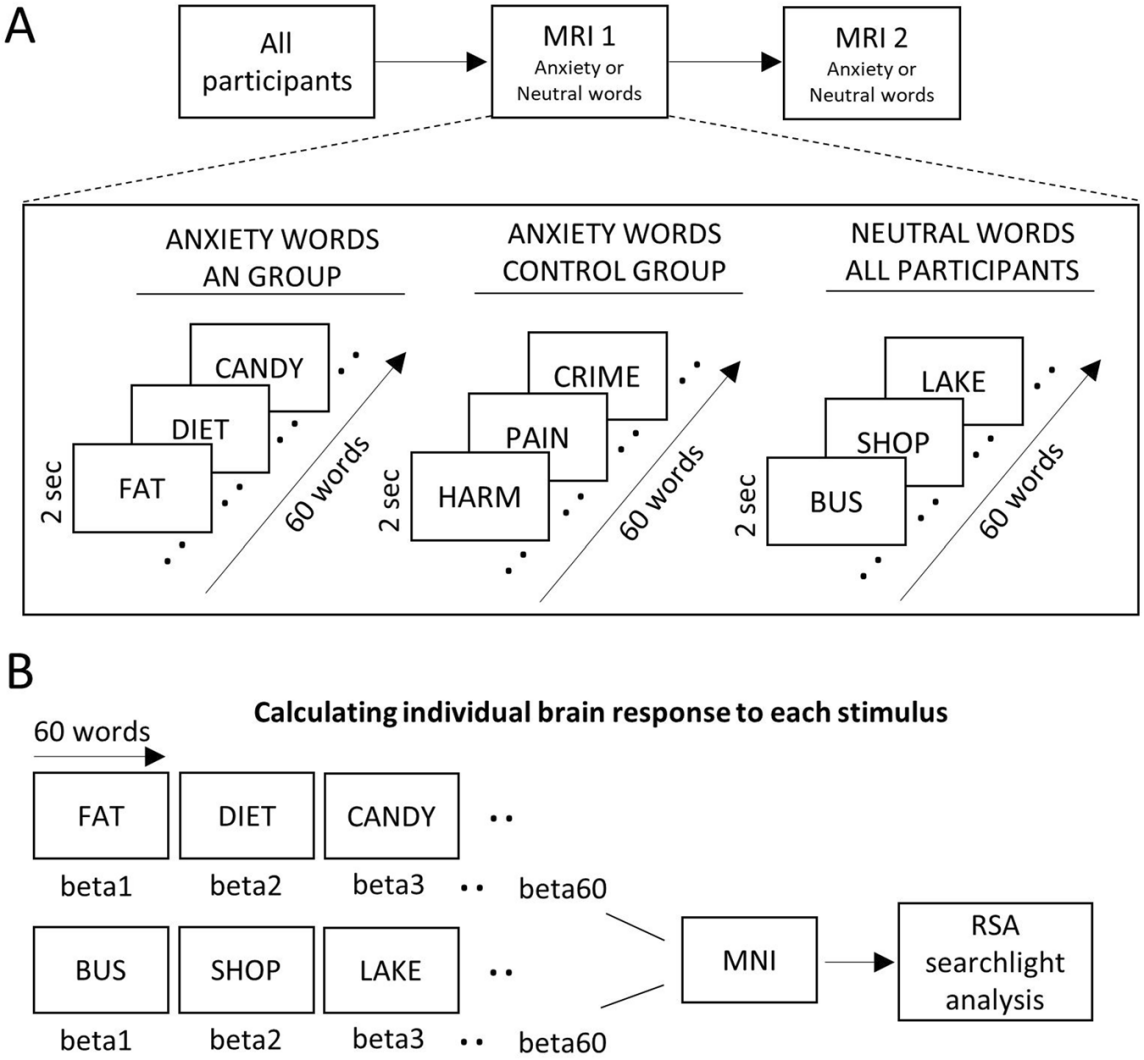
FMRI study design and brain response calculations. **A** FMRI Task Design: all participants underwent the same measurement regimen including two MRI sessions separated by two days. Anxiety or neutral words were presented either on day one or day two depending on the randomization. Participants with anorexia nervosa saw a set of personalized anxiety-eliciting words related to food while the control group saw a set of personalized general anxiety-eliciting words. During the neutral condition, participants were shown a set of personalized neutral stimuli. During each scanning day 60 words were presented, which were displayed for two seconds on the screen. (Of note, the word presentations shown here were part of a larger paradigm where people were also presented with additional stimuli to assess reward processing and reaction times.). **B** Calculation of Individual Brain Responses to Each Stimulus. Brain responses for each stimulus were calculated by using the beta-map derived from least-square-sum estimates. All 120 beta-maps (anxiety and neutral conditions) for each participant were normalized to standard Montreal Neurological Institute (MNI) space and forwarded to representational similarity analysis (RSA).

In general, all participants underwent a monetary reward/anxiety fMRI paradigm using a fast event-related design. (More details regarding the paradigm are found here [32]). The current analysis investigated the anxiety aspect of the study, where participants of both groups underwent the same scanning procedure and saw anxiety and neutral words (Fig. 1). Scans occurred at two time points, separated by two days. Participants were randomly assigned to participate either in the anxiety or neutral condition at scanning day 1, with the other condition occurring at scanning day 2. Anxiety-evoking words chosen based on personalized responses with the purpose of eliciting anxiety, hence people with AN saw words relevant for AN, while the control group was presented with a more generalized set of anxiety word stimuli. The 20 top-rated anxiety words during the initial presentation outside the scanner were then selected and used for the final paradigm. The 20 selected words (see word selection in the Supplement) were repeated three times during each scanning session, leading to 60 word presentations in total for each session. Each word was presented for 2 s on the screen.

### Data acquisition

Data were recorded with a 3 Tesla Siemens PRISMA scanner and a 64-channel coil. Echoplanar images (EPI) were acquired with a repetition time (TR) of 1000 ms, echo time (TE) of 33 ms, a flip angle of 80°, an isotropic voxel size of 2 mm^3^, a multiband acceleration factor of 5, field-of-view 208 mm, 487 volumes, and 60 slices. Structural MRI for registration consisted of a T1-weighted MPRAGE sequence with a TR of 2300 ms, TE of 2.99 ms, and an isotropic voxel dimension of 0.8 mm^3^.

### Anxiety circuit region of interest creation

The ROI mask (Fig. S1) was created by using probabilistic atlases and included core anxiety circuit regions [32–36]. The amygdala, anterior cingulate, insula, and medial prefrontal cortex were extracted from the Harvard-Oxford atlas with a 50% threshold, which was provided by the FSL software. In addition, the ventral tegmental area was delineated using the atlas from Pauli et al. [48] and for the bed nucleus of the stria terminalis an atlas by Theiss et al. [49] was used. As those latter regions are small, threshold values of 0.001 were chosen. All regions were then merged into a single ROI.

### Preprocessing

The anxiety and neutral fMRI run of each participant were first preprocessed within FSL (FMRIB’s Software Library, www.fmrib.ox.ac.uk/fsl) using FEAT (FMRI Expert Analysis Tool) Version 6.00, to carry out motion correction (MCFLIRT [50]) and temporal filtering. Both unsmoothed, native functional space fMRI runs from each participant were later forwarded to the RSA preprocessing pipeline. In addition, T1-weighted scans were brain extracted using BET [51] and later used for standard space registration using FLIRT [50, 52] before RSA was carried out (See supplement for detailed preprocessing steps).

### Representational similarity analysis

**Fig. 2:**
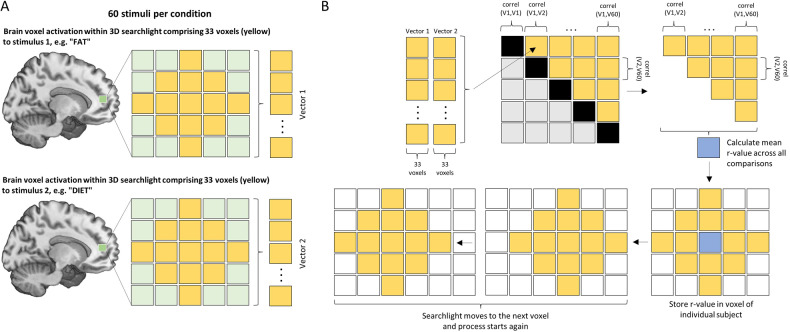
Schematic illustration of the representational similarity analysis approach. **A** Two examples of personalized stimuli (“FAT” & “DIET”) from the anxiety condition are depicted for a participant with anorexia nervosa. A 3-dimensional searchlight consisting of a 2-voxel-radius spherical ROI (33 voxels total) was placed in a single location in the brain to determine responses for each of the 60 stimuli during the first iteration. The activation pattern within the searchlight of the estimated beta-map was extracted, from which were created 60 unique vectors incorporating all the activation voxel values. **B** Pearson correlation coefficients were then calculated in a pair-wise fashion across all vectors (V1-V60) and stored in a similarity matrix. Values of the upper triangle were subsequently averaged and the single *r*-value was stored in a new brain map in the center of the same location of the searchlight. For the next iteration, the searchlight was then moved to the next voxel in the brain and the process was repeated until all voxels in the whole brain (or within the prespecified anxiety mask for the ROI analysis) were covered. This was carried out for the anxiety as well as for the neutral condition words, resulting in a brain map for each participant for both conditions.

Representational similarity analysis (RSA) [28] was conducted using custom MATLAB scripts. Within-anxiety word and within-neutral word RSA maps were calculated for each participant by correlating the single-trial beta-maps from the anxiety word presentations and the neutral word presentations respectively. We employed a searchlight mapping approach [37] within the anxiety ROI mask described above, as well as for the whole brain in an exploratory fashion. More specifically, RSA was conducted by first creating a set of 2-voxel-radius spherical ROIs centered over each voxel in the mask and then using the voxels contained within each mask to calculate the mean Pearson-correlation across all trials within each condition. This effort resulted in within-anxiety and within-neutral similarity maps for each participant, where each map’s voxel values represented the average r-value across all trials within a condition when the spherical ROI was centered on that voxel (for a schematic illustration see Fig. 2).

MATLAB scripts supporting this effort are available, open source at https://github.com/bbp-lab/seiger-2023

### Statistical analyses

#### Group analyses

We tested for differences in brain representational similarity to anxiety stimuli between groups using the anxiety word minus neutral word maps. We conducted anxiety-minus-neutral analyses to understand representational similarity differences between groups specifically for anxiety-provoking words, above and beyond any differences in representational similarity that might exist in response to viewing words in general.

##### Averaged ROI analysis

To compare multivariate responses averaged across the anxiety ROI mask, the RSA values of the anxiety mask were r-to-z transformed and averaged [31], leading to a single z-value per participant for each condition and ROI. We were also interested to what extent specific regions of the anxiety mask contribute to the results; as a planned follow-up analysis, each region comprising the overall anxiety circuit ROI was iteratively removed and scores were calculated again. This totaled 7 ROI analyses, done in R, for the anxiety-neutral condition, consisting of ANCOVAs to test between-group differences, with age and pubertal development scale (PDS) score as covariates and *P* < 0.05, two-tailed, as the statistical threshold.

##### Searchlight analyses: whole-brain and ROI

Difference maps were created by subtracting the final neutral word maps from the anxiety word maps for each participant. More specifically, this subtraction was applied to each r value in each voxel of the searchlight results brain map. We conducted a whole-brain analysis using a searchlight approach to detect differences between groups. Group differences between the AN group and controls were assessed with FSL, including age and PDS values as covariates of non-interest. A non-parametric permutation test with 5000 permutations and the threshold-free cluster enhancement (TFCE) method [53] using FSL’s Randomise was conducted, with a statistical threshold of *P* < 0.05, FWE-corrected. As a post hoc analysis, within-group responses were tested for anxiety minus neutral as well as for neutral minus anxiety conditions by using one-sample t-tests with covariates age and PDS for the whole-brain approach within FSL.

As an additional post-hoc exploratory analysis, this same analysis was repeated within the anxiety circuit ROI mask.

#### Associations with clinical variables

Correlations were carried out across all participants between the whole-brain searchlight maps and the HAM-A and, as an exploratory analysis, anxiety ratings after the scan. Pearson correlations were conducted separately for the anxiety and the neutral condition, expecting significant associations only for the anxiety trials. Whole-brain analyses were carried out again in FSL with Randomise and the correlations for the average values within the ROIs were calculated with R. As one participant from the AN group had a missing value for the anxiety rating after the scan, it was excluded from the analysis where the anxiety rating was used. To account for skewness and kurtosis of the data, RSA values were first square-root transformed before parametric statistics were performed. Furthermore, as many “0 ratings” were observed for the neutral condition and the anxiety ratings resulting in a highly skewed and non-normal distribution that could not be transformed, those values were excluded from the correlation analysis.

## Results

### Sample Characteristics

Twenty-five females with anorexia nervosa (14.6 ± 1.9 years) and 22 control participants (16.2 ± 1.8) were included in the final statistical analyses, after having excluded several participants due to: excessive motion (*n* = 3), wrong words or errors with the paradigm (7), missing or incomplete data (7) and binge-purge subtype (2). Mean ages between groups was significantly different (*t*-value = 2.99, *P*-value = 0.0045). In addition, the AN group had significantly higher HAM-A, DASS depression, Children’s Depression Rating Scale (CDRS) and self-reported anxiety scores (Table 1). PDS scores were significantly lower for the AN group (*P* < 0.001). No statistically significant differences were found for the DASS anxiety scale. Detailed information regarding the demographics and the psychometrics can be found in Table 1.

**Table 1:** Demographics and psychometrics.

	Participants with AN		*n*	Non-clinical controls		*n*	Statistics	*p*-value
	Mean ± SD	Median (IQR)		Mean ± SD	Median (IQR)			
Age	14.60 ± 1.94	15 (3)	25	16.23 ± 1.77	16 (3)	22	*t* = −2.99	0.0045
Education (years)	8.67 ± 2.10	8.5 (3)	24	10.36 ± 1.89	10.5 (3)	22	*W* = 140.5	0.0062
Proportion on medication	17/25	−	25	0/22	−	22	−	−
Duration of illness (months)	21.57 ± 18.66	12 (19)	21	−	−	−	−	−
BMI	18.14 ± 1.39	17.9 (1.24)	25	22.6 ± 2.91	22.6 (3.76)	22	*W* = 37	<0.001
BMI percentile	26.88 ± 17.43	27 (18)	25	62.36 ± 24.13	66 (32.25)	22	*t* = −5.83	<0.001
HAM-A	14.28 ± 7.8	11 (10)	25	7.23 ± 4.49	8 (6)	22	*t* = 3.85	<0.001
Anxiety rating (after anxiety run, 0–10)	5.38 ± 2.32	6 (3)	24	2.55 ± 1.77	3 (2)	22	*t* = 4.62	<0.001
Anxiety rating (after neutral run, 0–10)	4.80 ± 2.43	5 (3)	25	2.23 ± 2.11	2 (4)	22	*W* = 435	<0.001
DASS anxiety	14.17 ± 9.64	13 (10)	24	14.10 ± 6.21	14 (10)	21	*t* = 0.029	0.9769
DASS depression	19.91 ± 10.64	18 (16)	23	9.43 ± 6.87	10 (10)	21	*t* = 3.84	<0.001
CDRS total score	42.25 ± 18.56	39 (21.75)	24	23.09 ± 5.49	22 (4)	22	*W* = 470	<0.001
Pubertal development score	14.08 ± 4.41	16 (6)	24	17.85 ± 1.46	18 (2)	20	*W* = 100	<0.001
EDE score	3.50 ± 1.63	4.2 (2.63)	25	0.53 ± 0.74	0.2 (0.55)	22	*W* = 513.5	<0.001
YBC-EDS	24.38 ± 10.92	26 (14)	24	1.50 ± 3.16	0 (0)	22	*W* = 509	<0.001
Psychiatric comorbidities								
Major depressive disorder	8	–	–	–	–	–	–	–
Generalized anxiety disorder	12	–	–	–	–	–	–	–
Separation anxiety disorder	2	–	–	–	–	–	–	–
Specific phobia	1	–	–	–	–	–	–	–
Panic disorder	3	–	–	–	–	–	–	–
Agoraphobia	1	–	–	–	–	–	–	–
Generalized social phobia	4	–	–	–	–	–	–	–
Obsessive-compulsive disorder	5	–	–	–	–	–	–	–
Body dysmorphic disorder	1	–	–	–	–	–	–	–
No comorbidities	7	–	–	–	–	–	–	–

*AN* anorexia nervosa, *BMI* body mass index, *HAM-A* Hamilton Anxiety Rating Scale, *DASS* Depression Anxiety Stress Scales, *CDRS* Children’s Depression Rating Scale, *EDE* Eating Disorder Examination, *YBC-EDS* Yale-Brown-Cornell Eating Disorder Scale, *T-statistics* unpaired two-samples T-test, *W-statistics* unpaired two-samples Wilcoxon test; *n* number of available data per item, *SD* standard deviation, *IQR* interquartile range.

Mean with SD and median with IQR are presented.

### Between- and within-group results

The between-group searchlight analyses within the anxiety mask as well as for the whole-brain approach, carried out in FSL with Randomise, did not reveal statistically significant differences (no results survived *P* < 0.05, corrected). The ANCOVA conducted within *R* for the calculated single *z*-values for the anxiety circuit ROI also showed no significant differences between groups. More specifically, no significant effect of group on RSA outcome (anxiety minus neutral) after controlling for age and PDS for the anxiety circuit ROI mask was found, *F*(1,43) = 0.13, *P* = 0.72. Iteratively removing each specific region revealed the following results, which were also all non-significant: ACC removed: *F*(1,43) = 0.59, *P* = 0.45; Amygdala removed: *F*(1,43) = 0.11, *P* = 0.75; BNST removed: *F*(1,43) = 0.14, *P* = 0.71; Insula removed: *F*(1,43) = 0.11, *P* = 0.74; VTA removed: *F*(1,43) = 0.13, *P* = 0.72; mPFC removed: *F*(1,43) = 0.01, *P* = 0.92. These results suggest that the between-group results were not heavily influenced by any one region within the overall anxiety circuit mask.

**Fig. 3:**
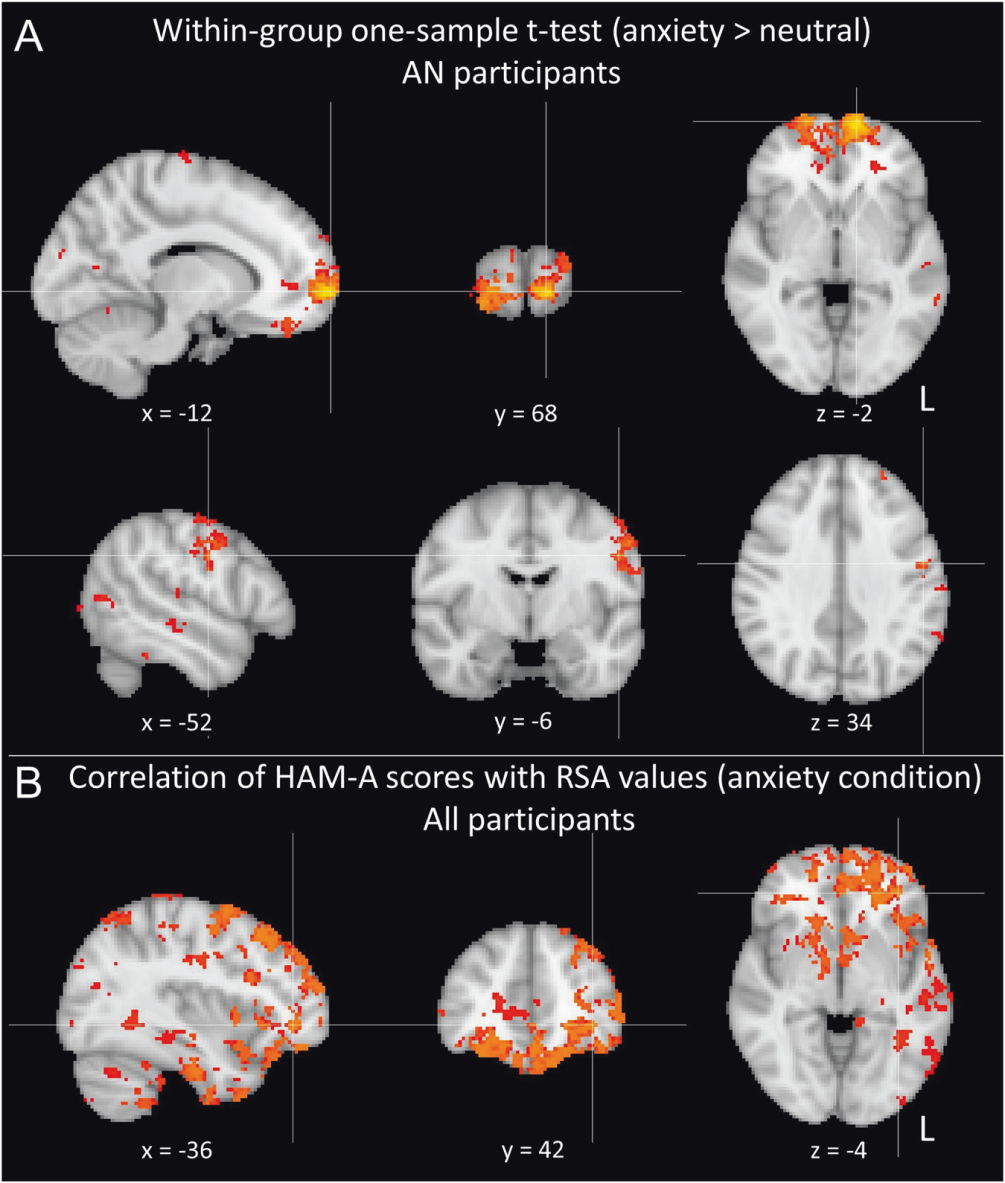
Representational similarity analysis results. **A** Within-group one-sample *t*-test for the anxiety minus neutral condition for participants with anorexia nervosa. **B** Results of the correlations of the HAM-A scores with the RSA values of the anxiety condition across all subjects. All results presented were *P* < 0.05, FWE-corrected.

Within-group results using one-sample t-tests and the covariates of age and PDS for the whole brain with Randomise showed significant results for the AN group for anxiety minus neutral, which were not observed for the control group. Results were predominantly found in prefrontal areas including, but not limited to, the medial orbitofrontal cortex, medial prefrontal cortex, dorsolateral prefrontal cortex, and the frontal pole, as well as other cortical regions including precentral and postcentral gyri, supramarginal gyrus, temporal fusiform gyrus, lingual gyrus, and intracalcarine cortex (see Fig. 3A and Tables 2 and S1).

**Table 2:** Representational similarity analysis cluster results.

Cluster #	T-max	Size (voxels)	*x*	*y*	*z*	Region
AN participants within-group one-sample *t*-test (anxiety > neutral)						
1	6.27	861	−12	68	−2	Frontal pole L
2	6.21	266	−52	−6	34	Precentral gyrus L
3	5.84	182	−68	−46	18	Supramarginal gyrus, posterior division L
4	5.72	108	−34	−28	68	Postcentral gyrus L
5	5.41	79	−66	−30	18	Supramarginal gyrus, anterior division L
6	5.33	45	10	−58	−6	Lingual gyrus R
7	5.22	28	8	−68	6	Intracalcarine cortex R
8	5.14	189	−34	−32	−22	Temporal fusiform cortex, posterior division L
9	4.91	28	−8	−68	14	Intracalcarine cortex L
10	4.79	4	38	−32	52	Postcentral gyrus R

Correlation of HAM-A scores with RSA values (anxiety cond.) across all participants						
1	6.27	573	46	−76	20	Lateral occipital cortex, superior division R
2	5.55	1425	−52	−12	−18	Middle temporal gyrus, posterior division L
3	5.45	1268	−28	28	46	Middle frontal gyrus L
4	5.36	160	−50	−46	20	Supramarginal gyrus, posterior division L
5	5.29	324	66	−38	36	Supramarginal gyrus, posterior division R
6	5.24	322	−26	6	−18	Frontal orbital cortex L
7	5.15	179	−48	−76	24	Lateral occipital cortex, superior division L
8	5.14	1890	−36	42	−4	Frontal pole L
9	5.05	42	22	4	40	Cerebral white matter R
10	5.04	43	12	−10	−2	Thalamus R

Results are presented for AN participants within-group for the anxiety minus neutral word condition (top) and for the associations between the HAM-A scores and the RSA values for all participants (bottom) (corresponding with the outcomes presented in Fig. 3). The top 10 clusters according to the maximum t-value (T-max) are presented. Regional brain labels are reported according with the Harvard-Oxford atlas.

### Associations with clinical variables

The whole-brain FSL correlational analysis revealed a significant positive association between the HAM-A scores and the RSA values for the anxiety condition (*P* < 0.05, corrected). Results were present in cortical and subcortical regions including a large prefrontal cluster with peak magnitude in the frontal pole, as well as clusters in the occipital cortex, temporal cortex, supramarginal gyrus, frontal orbital cortex, thalamus, and cerebellum (Fig. 3B and Tables 2 and S1) (although not emerging as separate clusters, significant clusters also spanned the amygdala and hippocampus). No significant results were observed for the neutral condition.

No significant results were observed for the anxiety ratings after the scan. In addition, no negative correlations between HAM-A or anxiety ratings after the scan and RSA values were statistically significant.

**Fig. 4:**
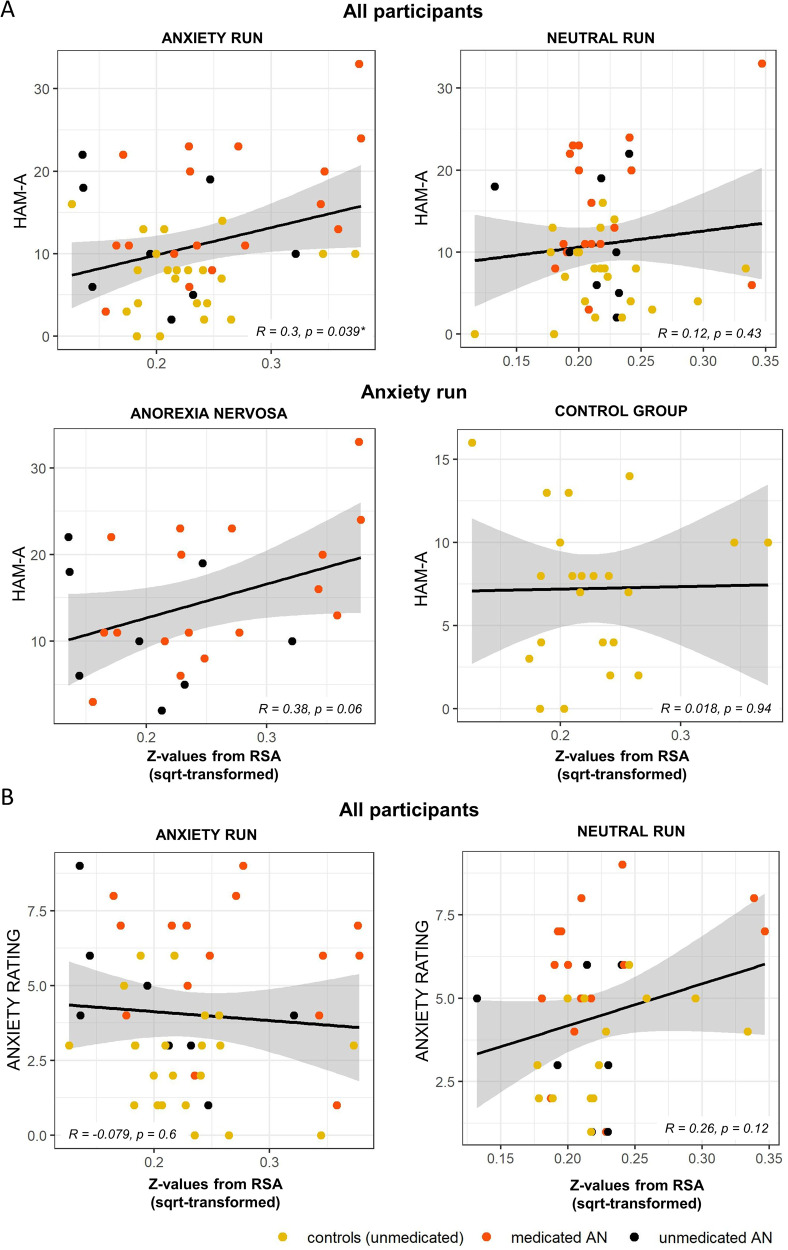
Relationships between anxiety ratings and representational similarity (ROI approach). **A** Correlations of Hamilton Anxiety Rating Scale (HAM-A) values and the Representational Similarity Analysis (RSA) metrics for the anxiety and neutral run. **B** Correlations of the self-reported anxiety ratings (Likert Scale: 0–10) obtained directly after the scan and the RSA metrics for the anxiety and neutral run. All RSA values on the x-axis were square-root transformed to account for skewness and kurtosis. Asterisk indicates significant result (*p* < 0.05), while colors indicate control subjects as well as medicated and unmedicated patients.

The association (Pearson correlation) between the anxiety circuit ROI RSA values and the HAM-A scores across participants showed a significant positive correlation for the anxiety condition (*r* = 0.30, *P* = 0.04), which was not observed for the neutral condition (*r* = 0.12, *P* = 0.43). Investigating each group separately, results suggested that the correlation was mainly driven by the participants with anorexia (*r* = 0.38, *P* = 0.06), while the control participants showed no associations (*r* = 0.02, *P* = 0.94) (Fig. 4A). However, correlations between the groups were not significantly different (Fisher’s z transformation: *z* = 1.22, *P* = 0.11). The same analysis was conducted for the anxiety ratings after the scan; no significant association were observed for the anxiety (*r* = −0.08, *P* = 0.60) nor for the neutral condition (*r* = 0.26, *P* = 0.12 (Fig. 4B)). Furthermore, an additional exploratory analysis with DASS-Anxiety scores has been carried out, which can be found in the supplementary material (see also Figs. S2 and S3 and Table S2).

## Discussion

This investigation examined multivariate neural representations of anxiety in adolescents with anorexia nervosa and non-clinical adolescents with mild anxiety. As hypothesized, there were significant associations between anxiety symptoms and representational similarity across participants in anxiety circuit regions, as well as in widespread cortical and subcortical regions. These were driven primarily by the AN group, suggesting that they exhibit strong relationships between anxiety symptoms and consistency of response patterns to anxiety-provoking stimuli. Contrary to our predictions, there were no significant group differences in response patterns to anxiety stimuli in the averaged representational similarity values within the anxiety circuit. Further, neither the voxel-wise searchlight analysis of the whole brain nor the exploratory searchlight analysis within the anxiety circuit ROI revealed significant group differences. Non-significant group differences may have been due to the fact that controls were selected based on having higher anxiety than population norms, as the study was designed to dimensionalize anxiety across clinical and non-clinical populations.

Nevertheless, a follow-up within-group whole brain voxel-wise analysis revealed significant within-group results in the AN group, which were not found in the control participants. There was significantly higher representational similarity in response to anxiety words over neutral words in multiple cortical areas in the AN group, including prefrontal cortical regions, precentral- and postcentral gyrus, supramarginal gyrus, temporal fusiform gyrus, lingual gyrus, and intracalcarine cortex. This suggests a more consistent, “orchestrated” response in AN to anxiety-provoking stimuli compared with responses to neutral stimuli.

Regarding the prefrontal areas showing strong representational similarity in AN, high degrees of prefrontal activation in response to food stimuli have been shown in prior studies in young adults with AN using univariate approaches, including greater activations in people with eating disorders in the medial orbitofrontal cortex [19], and greater dorsolateral prefrontal cortex activations for those with restricting type AN [21], in contrast to controls. In these studies, these prefrontal regions’ activations were interpreted as possibly due to cognitive biases related to food and associated urges to suppress or control thoughts related to food intake, which may also be related to the compulsive features of AN. This observation is also in line with the concept of ‘habit learning’, where stimulus-response schemes are automatically executed. Studies showed that mainly striatal brain circuits are activated, which are, amongst others, also regulated by prefrontal brain regions as shown in our investigation [54–56].

A specific prefrontal region showing higher representational similarity for anxiety compared with neutral words in those with AN in the current study was the medial prefrontal cortex. This region has been shown to be responsive to reward and the affective value of a stimulus [2], In addition, it has strong subcortical connections and is involved in attention and inhibitory control [57]. Specifically, the ventromedial prefrontal cortex vmPFC is involved in emotional regulation and processing, and decision making [58], and modulates activity of limbic regions such as the amygdala [59]. Thus, it is possible that when those with AN find themselves in a state of anxiety or discomfort elicited by words related to food, weight, body image, etc., the medial prefrontal cortex may be consistently and stereotypically activated to enact downregulation of limbic circuits. Also observed were high degrees of representational similarity in the medial frontal pole. The medial frontal pole is connected to nodes of the default mode network [60, 61] known for its involvement in internally focused, self-relevant cognitions [62, 63]. This raises the possibility that consistent internally focused cognitive processing may have occurred during the task during exposure to disorder-relevant anxiety words in AN. This is supported by the fact that participants were presented personalized words, although this remains speculation as the degree that their thinking was self-focused during the task was not assessed.

The correlation analysis between HAM-A scores and RSA values elicited by the anxiety words showed a positive relationship within the anxiety circuit mask as well as in a set of distributed regions from the whole-brain voxel-wise analysis. This suggests a more consistent brain response in people with higher anxiety symptomology in regions explicitly involved in anxiety processing as well as outside of classical anxiety circuits. Consistent activation patterns to anxiety-provoking stimuli in those with higher anxiety could represent more persistent, conditioned neural representation of anxiety or threat, as has been previously demonstrated in a conditioning experiment using RSA [18]. In other words, those with higher anxiety could have more persistent Pavlovian conditioning linking experiences, situations, physical items (food), etc. and the words that represent them. If so, a possible explanation for persistence is a failure of fear extinction, a phenomenon that has been demonstrated in obsessive-compulsive disorder, which shares behavioral phenotypes with AN and is commonly comorbid [6, 16, 64–66]. For example, some with AN struggle with fear and aversion to reminders of food items that they once avoided due to associations with the risk of weight gain such as high calorie desserts; seeing these items on a menu may continue to evoke fear and make it difficult to eat in restaurants, even after experiencing this situation many times (after which one might ordinarily be expected to have desensitized to it) as well as consciously knowing that the fear is not rational.

In the whole-brain analysis, positive associations between anxiety symptoms and representational similarity were found in prefrontal regions (including the medial prefrontal cortex and frontal pole – see interpretations above), and in occipital, temporal, parietal, cerebellar, and subcortical areas such as the thalamus. These associations partially overlap with the prespecified core anxiety mask as well as with regions of extended anxiety circuitry where we predicted higher RSA values in people with AN in contrast to control participants. Occipital regions and the thalamus are involved in the integration of sensory information and have shown greater activity in patients with anxiety compared to healthy controls when confronted with threat-eliciting stimuli [42]. In the context of the conditioned fear learning, a conditioning study in a large transdiagnostic anxiety/depression cohort showed involvement of a distributed network comprising regions, amongst others, that included the middle frontal gyrus and the cerebellum [67], as was found in the current study.

Significant correlations were not found, on the other hand, between representational similarity and state anxiety. Hence, anxiety symptoms (that is, more enduring symptoms over the previous week, as assessed by the HAM-A) rather than current state anxiety are linked to consistency of brain responses. In addition, no significant *negative* correlations were observed for anxiety words; thus, higher anxiety symptoms were not associated with lower consistency of responses in any brain regions. Furthermore, and as expected, no significant positive or negative correlations were found for the neutral words control analysis, supporting the specificity of anxiety-related response patterns.

Several neuroimaging studies with AN participants involving food stimuli have been conducted, with variable findings across studies (see “Introduction” section for an overview), which could partially be explained by different paradigms, stimuli, and methodological approaches. However, limbic regions such as the amygdala and the anterior cingulate cortex as well as the insula have been frequently reported to be involved in processing those stimuli related to anxiety, fear, or to uncomfortable feelings in AN or in eating disorders in general [19, 20, 22–24]. The observation in the current study that representational similarity was not significant within the AN group in these systems (although in limbic/paralimbic regions representational similarity was associated with severity of anxiety symptoms) could have been attributed to less consistent responses due to engagement of fronto-limbic top-down modulation. Importantly, in contrast to those prior studies, we used a multivariate approach to gain information about distributed patterns within the data, which revealed regions outside of anxiety circuits; these could have been absent or inconsistent across previous studies in AN using traditional univariate approaches, which only yield peak activations [30].

The AN sample was scanned after being treated intensively in an inpatient, residential, or partial hospitalization program where they were partially or fully weight restored. Yet, full neurotransmitter function recovery may be delayed after malnutrition states [68]. In one study, elevated reward response was observed after weight restoration, implicating possible persistent (or, preexisting) dopaminergic dysfunction [69]. Even in recovered individuals with AN, abnormal dopaminergic D2/D3 receptor binding was found in the antero-ventral striatum [70]. Persistent dopaminergic dysfunction may be relevant for fear extinction; prediction errors that arise when an expected aversive stimulus does not occur are encoded as dopaminergic signals from the ventral tegmental area to the ventral striatum [71]. In turn, this engages fear extinction circuitry, in particular the vmPFC (facilitated by D2 receptors [72]) and amygdala [73]. Persistent dopaminergic dysfunction may therefore affect this fear extinction pathway. Alterations of serotonergic functioning have also been observed to persist after recovery in AN (reviewed in [68]), which, along with dopaminergic dysfunction, may affect mood, anxiety, and obsessive/compulsive symptoms, potentially having an indirect impact on fear responses. Thus, delayed recovery from malnutritional states of dopaminergic or serotonergic systems may directly or indirectly affect fear responses. Yet, in the current sample, these were likely quite variable across participants due to different durations of time between when they were in malnutrition states and when they were scanned, in part due to criteria for discharge not solely being based on weight or nutritional status.

The results of the current study have potential clinical implications. Adolescents with strong anxiety symptoms – and, in particular, those with AN with strong anxiety – show highly consistent patterns of neural activity for anxiety-provoking stimuli. This could manifest clinically as invariantly reactive fear responses to one’s environment, as opposed to more adaptive, nuanced patterns of responding according to different situations. For those with AN with strong anxiety, this could mean high distress in situations when their fear is evoked by (conditioned) reminders of weight, food, bodies, etc. This could subsequently result in persistent avoidant patterns, contributing to persistent inability to achieve or maintain a healthy body weight along with other functional impediments. These results may have implications not only for understanding behavioral phenomenology but also highlight potential challenges for treatments addressing conditioned fear responses with systematic desensitization (a typical approach in CBT) and inform additional research into possible alternative approaches to address conditioned fear such as counterconditioning [74].

There are several limitations to consider. Firstly, the population studied was relatively homogenous in terms of age and gender, namely, all young adolescent female participants. This should be taken into consideration in interpreting the results and if/how our findings are applicable to other populations. However, there was a statistically significant age difference between the groups, with a higher age for the non-clinical control cohort due to recruiting problems caused by COVID-19. This was accounted for statistically by including age as a covariate. Conversely, we did not explicitly control for BMI as it is colinear with the diagnosis of AN (and thus colinear with an effect of interest) and also related to the PDS scores, which were used as a covariate. The participants were not in the starvation state, thus extremes of BMI were not likely affecting results. Nevertheless, potential effects related to BMI thus cannot be ruled out and future studies that, for example, use a control group without AN but matched on BMI could help shed light on this issue. The sample size is an additional limitation, which may have limited our ability to detect group differences and did not permit a sub-analysis of medicated (*n* = 17)/unmedicated (*n* = 8) participants. People with AN often experience other symptoms, in addition to anxiety, such as depression and eating disorder-related obsessions and compulsions. This was also observed in our sample, where relatively strong correlations between anxiety symptoms (HAM-A) and depression scores (CDRS) (*r* = 0.6, *p* = 0.002) and obsessions/compulsions (YBC-EDS) (*r* = 0.44, *p* = 0.03) were observed. As a limitation, the specific contributions of these other symptoms to modulating the observed response patterns in the patient cohort are difficult to discern as they interact with, and therefore are not independent from, anxiety. A similar limitation pertains to comorbidities, the combination of which and the sample sizes of subgroups precluded additional analyses. Further, we asked participants *after* the scan to rate their anxiety, to approximate state anxiety during the experiment; however, anxiety that they may have experienced during the task when viewing the words could have resolved at that point. We did not assess their subjective anxiety during the middle of the scan, as doing so would likely have a modulatory effect on emotional experiences and related brain activation. In addition, only disorder-specific anxiogenic stimuli were used in this paradigm. Response patterns to other anxiogenic stimuli such as those related to change, uncertainty or risk, e.g., might also play an important role in those with AN.

In sum, these results suggest that anxiety symptoms are associated with consistent brain responses within- and outside of classical anxiety circuits, particularly in adolescents with AN. In addition, adolescents with AN consistently engage distributed systems in response to anxiety-provoking stimuli, particularly prefrontal regions, which may be involved in down-regulating limbic regions. This may reflect learned and automatic brain response in people with AN to anxiety-eliciting stimuli regarding eating, food intake, and body image. The results from this representational similarity multivariate analysis have implications for future research, providing insight into both the consistency and distributed patterns of neural responses, including those that may be the result of fear-conditioning to environmental stimuli.

### Supplementary information

Supplemental material

Supplement legends

Figure S1

Figure S2

Figure S3

Table S1

Table S2

## Data Availability

The data that support the findings of this study are openly available in the NIMH Data Archive at https://nda.nih.gov/, collection ID 2565
