# Supplementary material for: Neural representations of anxiety in adolescents with anorexia nervosa: a multivariate approach
Source: Transl Psychiatry. 2023 Aug 15;13:283. doi: 10.1038/s41398-023-02581-5 (PMC10427677; doi:10.1038/s41398-023-02581-5)
Supplement: Supplementary file 1 — Supplemental material [file 41398_2023_2581_MOESM1_ESM.docx]

**Participants**

Study participants were recruited from local eating disorder treatment centres and by advertisements and flyers. Treatment in these programs comprised psychotherapy and dietary monitoring, as well as pharmacotherapy for some. As a result of this treatment, participants with AN were partially or fully weight-restored. For inclusion in the study, participants with AN were either free of medication or were on stable doses of a selective serotonin intake inhibitor or a serotonin and norepinephrine reuptake inhibitor during the previous 8 weeks before enrollment. Exclusion criteria for participants with AN comprised lifetime Axis I bipolar disorder, lifetime psychotic disorders, lifetime attention deficit hyperactivity disorder, or current post-traumatic stress disorder. Further exclusion criteria for both groups comprised current substance abuse and/or dependence, pathological gambling, current medical or neurological disorders, pregnancy, and MRI contraindications such as metal implants or electronic devices. We chose sample sizes based on the primary analysis outcome of interest: to detect significant relationships between brain activation patterns in blood oxygen level (BOLD) % signal change in reward systems and change in adjusted BMI post-treatment in AN participants. The current analysis represents a secondary data analysis.

**Clinical evaluations**

Clinical evaluations were performed by licensed clinician team members with expertise in AN. Screening was conducted with the Mini-International Neuropsychiatric Interview (MINI KID 7.0.2) ^1^. AN symptom severity was assessed using the Eating Disorder Examination (EDE) ^2^ and the Yale-Brown-Cornell Eating Disorder Scale (YBC-EDS) ^3^. Depression severity was assessed with the Children’s Depression Rating Scale^TM^, Revised (CDRS^TM^-R) ^4^ and DASS-21 depression subscale. Strength of motivation to follow life’s goals were assessed with the Behavioral Inhibition System/Behavioral Activation System (BIS/BAS) ^5^ and body dysmorphic disorder (BDD) was assessed with the BDD Diagnostic Module – Adolescent version ^6^.

**Word selection for the fMRI experiment**

Prior to scanning, all participants with AN and controls were shown 150 words on a computer screen, which they rated on a scale from 1-9 (9 being the highest) by answering the question, “How anxious does this word make you feel?”. The words were taken from three different lists: 50 anorexia-anxiety words (e.g. fat, diet), 50 general-anxiety words (e.g. harm, pain) and 50 neutral words (e.g. bus, shop) adapted from prior studies ^7–12^. Words were picked from the created three lists for presentation to participants in a randomized order. Each participant’s top 20 words with the highest anxiety ratings were then chosen for the anxiety run. For the AN group, only the anorexia-anxiety words were presented and for the non-clinical comparisons only the general-anxiety words were presented. The neutral words for both groups were chosen from the neutral list, which were the 20 ranked as least anxiety-provoking. Further, we ensured that the overall number of syllables did not significantly differ between the two lists for each participant; if significant differences were present, the next-highest (anxiety) or next-lowest (neutral) word was selected until there were no significant differences.

**Calculation of beta values and additional preprocessing**

Preprocessed time series data in native space was forwarded to AFNI ^13^ and deconvolved using the 3dDeconvolve command. Least-square-sum estimates of beta values using 3dLSS were conducted for each word presentation stimulus within the respective run. In addition to the word presentations of interest, all other events presented during the paradigm were included for modelling, (although these were not analyzed in this investigation). The 120 single-trial beta-maps from each participant, calculated from the 60 anxiety word and the 60 neutral word presentations, were brought into MNI standard space by using the transformation matrices calculated before within FSL and were subsequently forwarded to the RSA pipeline. Time points with movement outliers, according to FSL's motion outlier tool (fsl_motion_outliers), where the default threshold was used, were excluded from further analysis according to the DVARS metric ^14^.

**Missing PDS scores**

Three participants had missing values for the PDS data. For further analysis, those missing data points were imputed by taking the mean value of other participants within the same age range (±1 year) within the respective group.

**Associations between DASS-Anxiety scores and RSA metrics**

Next to the HAM-A and the anxiety ratings, we conducted exploratory analyses to test associations between the DASS-Anxiety scale and RSA values. Comparing the HAM-A to the DASS-Anxiety subscale ROI results, both scales capture similar neural patterns of responses in these regions (Figure S3).

The whole brain searchlight results, however, revealed differences in prefrontal RSA patterns between the two scales, especially in the OFC and frontal pole; while for the HAM-A strong correlations were found in those prefrontal regions, they were absent for the DASS-Anxiety scale. On the other hand, strong associations were found for the DASS-Anxiety scores and RSA values in the insula, post- and precentral gyrus and the hippocampus among other areas (see Figure S2 and Table S2 for detailed results).

**References**

1 Sheehan D V., Sheehan KH, Shytle RD, Janavs J, Bannon Y, Rogers JE *et al.* Reliability and validity of the mini international neuropsychiatric interview for children and adolescents (MINI-KID). *J Clin Psychiatry* 2010; **71**: 313–326.

2 Carter JC, Stewart DA, Fairburn CG. Eating disorder examination questionnaire: Norms for young adolescent girls. *Behav Res Ther* 2001; **39**: 625–632.

3 Jordan J, Joyce PR, Carter FA, McIntosh VVW, Luty SE, McKenzie JM *et al.* The Yale-Brown-Cornell eating disorder scale in women with anorexia nervosa: What is it measuring? *Int J Eat Disord* 2009; **42**: 267–274.

4 Overholser JC, Brinkman DC, Lehnert KL, Ricciardi AM. Children’s Depression Rating Scale-Revised: Development of a short form. *J Clin Child Psychol* 1995; **24**: 443–452.

5 Carver CS, White TL. Behavioral Inhibition, Behavioral Activation, and Affective Responses to Impending Reward and Punishment: The BIS/BAS Scales. *J Pers Soc Psychol* 1994; **67**: 319–333.

6 Phillips KA, Atala KD, Pope HG. Diagnostic instruments for body dysmorphic disorder. In: *New Research Program and Abstracts, American Psychiatric Association 148th Annual Meeting*. Miami, 1995, p 157.

7 Redgrave GW, Bakker A, Bello NT, Caffo BS, Coughlin JW, Guarda AS *et al.* Differential brain activation in anorexia nervosa to Fat and Thin words during a Stroop task. *Neuroreport* 2008; **19**: 1181–1185.

8 Miyake Y, Okamoto Y, Onoda K, Shirao N, Okamoto Y, Otagaki Y *et al.* Neural processing of negative word stimuli concerning body image in patients with eating disorders: An fMRI study. *Neuroimage* 2010; **50**: 1333–1339.

9 Shirao N, Okamoto Y, Okada G, Okamoto Y, Yamawaki S. Temporomesial activation in young females associated with unpleasant words concerning body image. *Neuropsychobiology* 2003; **48**: 136–142.

10 Mathews A, Mogg K, May J, Eysenck M. Implicit and Explicit Memory Bias in Anxiety. *J Abnorm Psychol* 1989; **98**: 236–240.

11 Dindo L, Fowles DC. The skin conductance orienting response to semantic stimuli: Significance can be independent of arousal. *Psychophysiology* 2008; **45**: 111–118.

12 Isenberg N, Silbersweig D, Engelien A, Emmerich S, Malavade K, Beattie B *et al.* Linguistic threat activates the human amygdala. *Proc Natl Acad Sci U S A* 1999; **96**: 10456–10459.

13 Cox RW. AFNI: Software for analysis and visualization of functional magnetic resonance neuroimages. *Comput Biomed Res* 1996; **29**. doi:10.1006/cbmr.1996.0014.

14 Power JD, Barnes KA, Snyder AZ, Schlaggar BL, Petersen SE. Spurious but systematic correlations in functional connectivity MRI networks arise from subject motion. *Neuroimage* 2012; **59**: 2142–2154.
