# Supplementary material for: Neural representations of anxiety in adolescents with anorexia nervosa: a multivariate approach
Source: Transl Psychiatry. 2023 Aug 15;13:283. doi: 10.1038/s41398-023-02581-5 (PMC10427677; doi:10.1038/s41398-023-02581-5)
Supplement: Supplementary file 2 — Supplement legends [file 41398_2023_2581_MOESM2_ESM.docx]

**Figure S1:** Regions included in the anxiety circuit mask. Abbreviations: ACC: Anterior cingulate cortex; BNST: Bed nucleus of the stria terminalis; MPFC: Medial prefrontal cortex; VTA: Ventral tegmental area.

**Figure S2:** Results of the associations between the Depression Anxiety Stress Scale (DASS) (Anxiety Subscale) scores and the RSA values of the anxiety condition across all subjects. All results presented were *P*<.05*,* FWE-corrected.

**Figure S3:** Correlations of Depression Anxiety Stress Scale (DASS) values (Anxiety Subscale) and the Representational Similarity Analysis (RSA) metrics for the anxiety and neutral run for all participants (ROI Approach). RSA values on the x-axis were square-root transformed to account for skewness and kurtosis. Colors indicate control subjects as well as medicated and unmedicated patients.

**Table S1:** Representational Similarity Analysis Cluster Results (Continuation of Table 2). Results are presented for AN participants within-group for the anxiety minus neutral word condition (top) and for the correlations between the HAM-A scores and the RSA values for all participants (bottom) (corresponding with the outcomes presented in Figure 3). Clusters from number 11 onwards (to cluster number 60) according to the maximum t-value (T-max) are presented. Regional brain labels are reported according with the Harvard-Oxford atlas.

**Table S2:** Representational Similarity Analysis Cluster Results. Results are presented for the associations between the DASS-Anxiety scores and the RSA values for all participants (corresponding with the outcomes presented in Figure S2). Clusters from number 1 onwards to cluster number 60 according to the maximum t-value (T-max) are presented. Regional brain labels are reported according with the Harvard-Oxford atlas.
