# Supplementary figures and images for: Neural representations of anxiety in adolescents with anorexia nervosa: a multivariate approach
Source: Transl Psychiatry. 2023 Aug 15;13:283. doi: 10.1038/s41398-023-02581-5 (PMC10427677; doi:10.1038/s41398-023-02581-5)

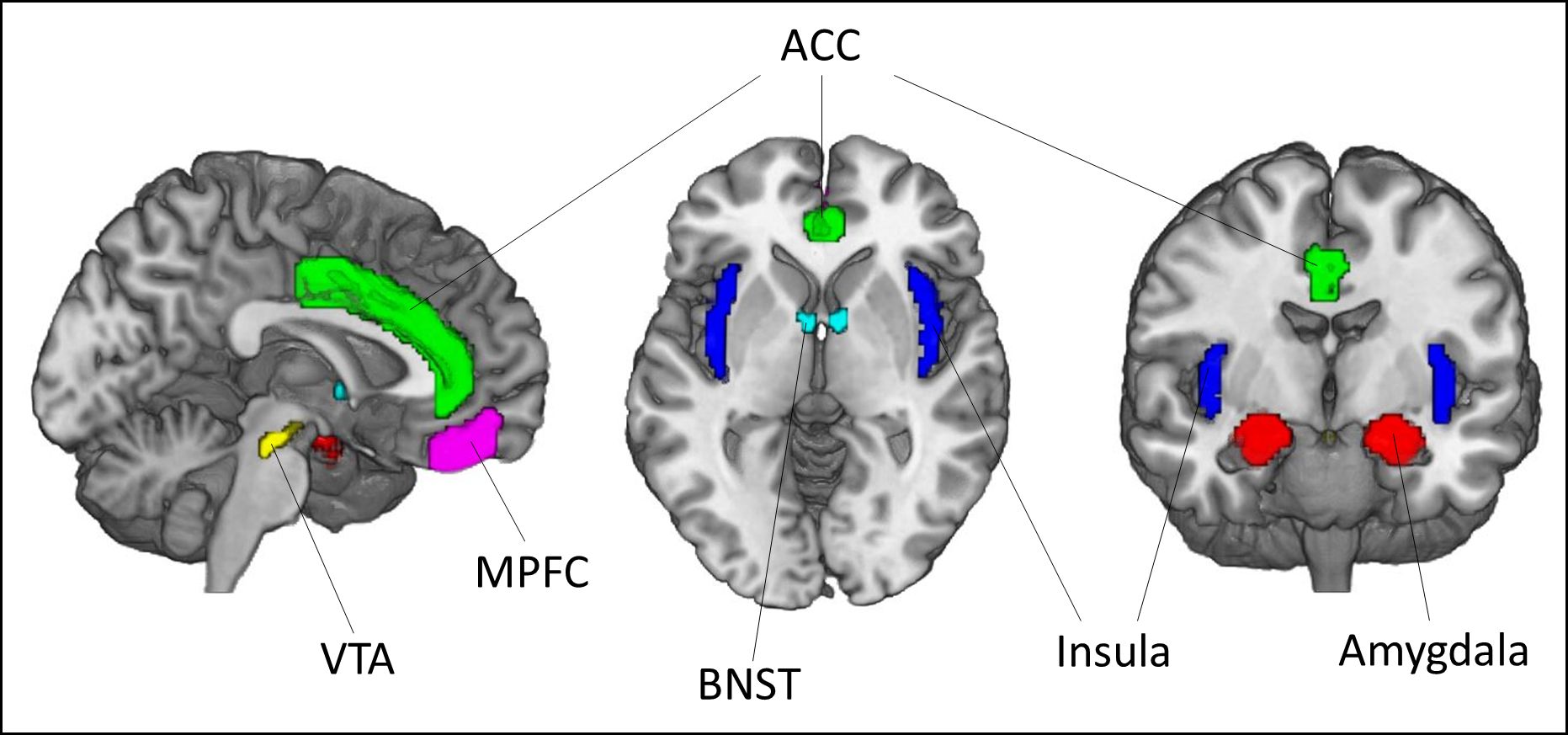

Supplement: Supplementary file 3 — Figure S1 [file 41398_2023_2581_MOESM3_ESM.jpg]

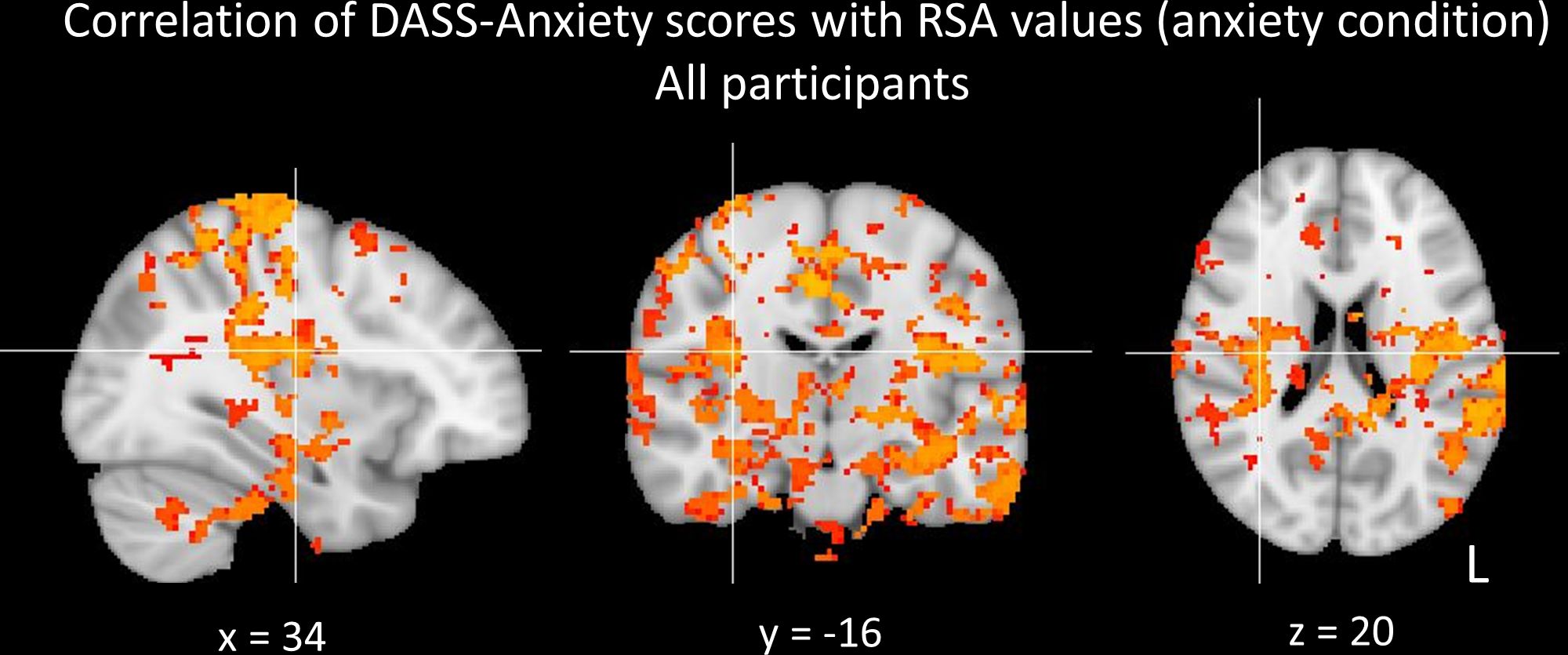

Supplement: Supplementary file 4 — Figure S2 [file 41398_2023_2581_MOESM4_ESM.jpg]

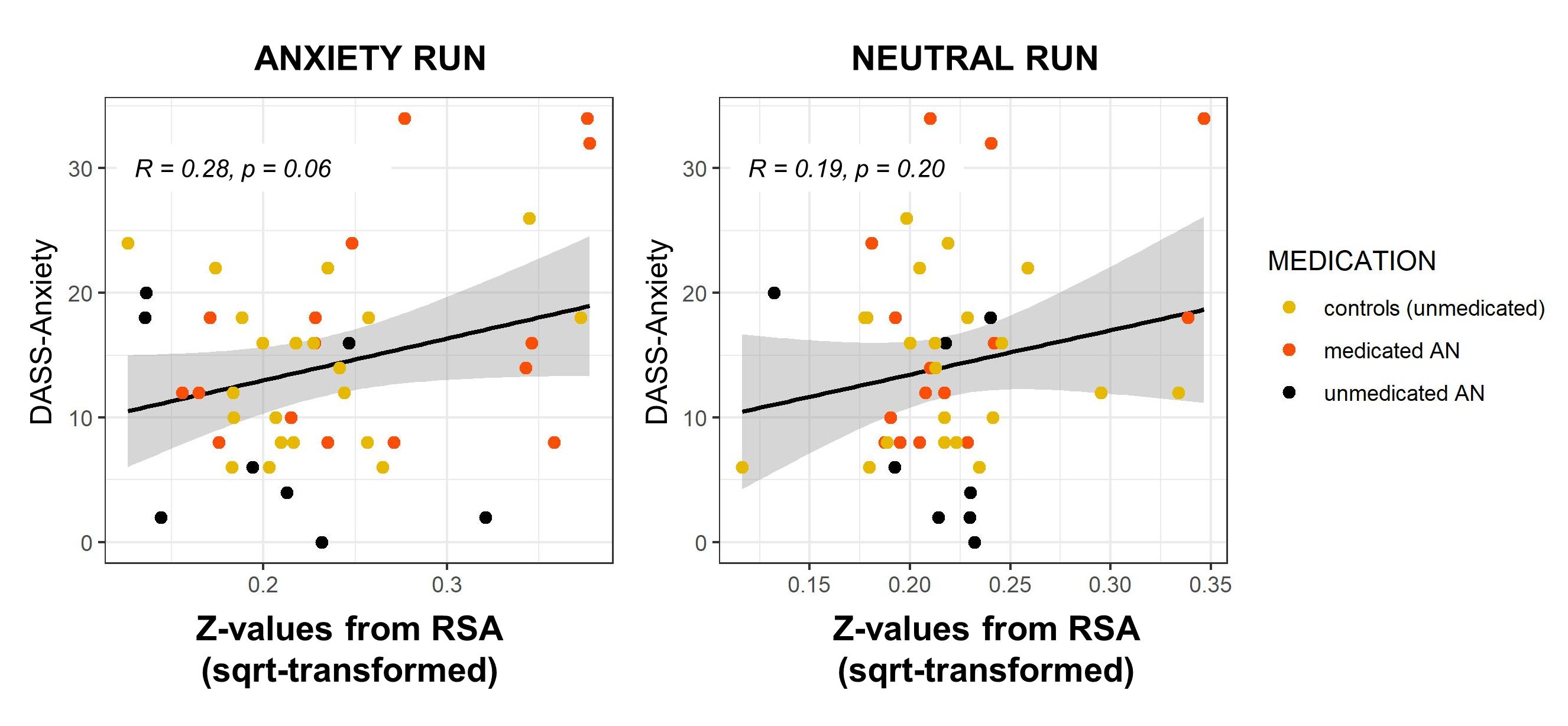

Supplement: Supplementary file 5 — Figure S3 [file 41398_2023_2581_MOESM5_ESM.jpg]
